# You Shall Not Pass: Root Vacuoles as a Symplastic Checkpoint for Metal Translocation to Shoots and Possible Application to Grain Nutritional Quality

**DOI:** 10.3389/fpls.2018.00412

**Published:** 2018-04-03

**Authors:** Felipe K. Ricachenevsky, Artur T. de Araújo Junior, Janette P. Fett, Raul A. Sperotto

**Affiliations:** ^1^Departamento de Biologia, Programa de Pós-Graduação em Agrobiologia, Universidade Federal de Santa Maria, Santa Maria, Brazil; ^2^Programa de Pós-Graduação em Biologia Celular e Molecular, Universidade Federal do Rio Grande do Sul, Porto Alegre, Brazil; ^3^Departamento de Botânica, Universidade Federal do Rio Grande do Sul, Porto Alegre, Brazil; ^4^Centro de Ciências Biológicas e da Saúde, Programa de Pós-Graduação em Biotecnologia, Universidade do Vale do Taquari – UNIVATES, Lajeado, Brazil

**Keywords:** biofortification, nutritional quality, vacuolar transport, metals, ionome

## Abstract

Plant nutrient uptake is performed mostly by roots, which have to acquire nutrients while avoiding excessive amounts of essential and toxic elements. Apoplastic barriers such as the casparian strip and suberin deposition block free diffusion from the rhizosphere into the xylem, making selective plasma membrane transporters able to control elemental influx into the root symplast, efflux into the xylem and therefore shoot translocation. Additionally, transporters localized to the tonoplast of root cells have been demonstrated to regulate the shoot ionome, and may be important for seed elemental translocation. Here we review the role of vacuolar transporters in the detoxification of elements such as zinc (Zn), manganese (Mn), cadmium (Cd), cobalt (Co) and nickel (Ni) that are co-transported with iron (Fe) during the Fe deficiency response in *Arabidopsis thaliana*, and the possible conservation of this mechanism in rice (*Oryza sativa*). We also discuss the evidence that vacuolar transporters are linked to natural variation in shoot ionome in Arabidopsis and rice, indicating that vacuolar storage might be amenable to genetic engineering without strong phenotypical changes. Finally, we discuss the possible use of root’s vacuolar transporters to increase the nutritional quality of crop grains.

## Root Apoplastic and Symplastic Control of Metal Uptake

**FIGURE 1:**
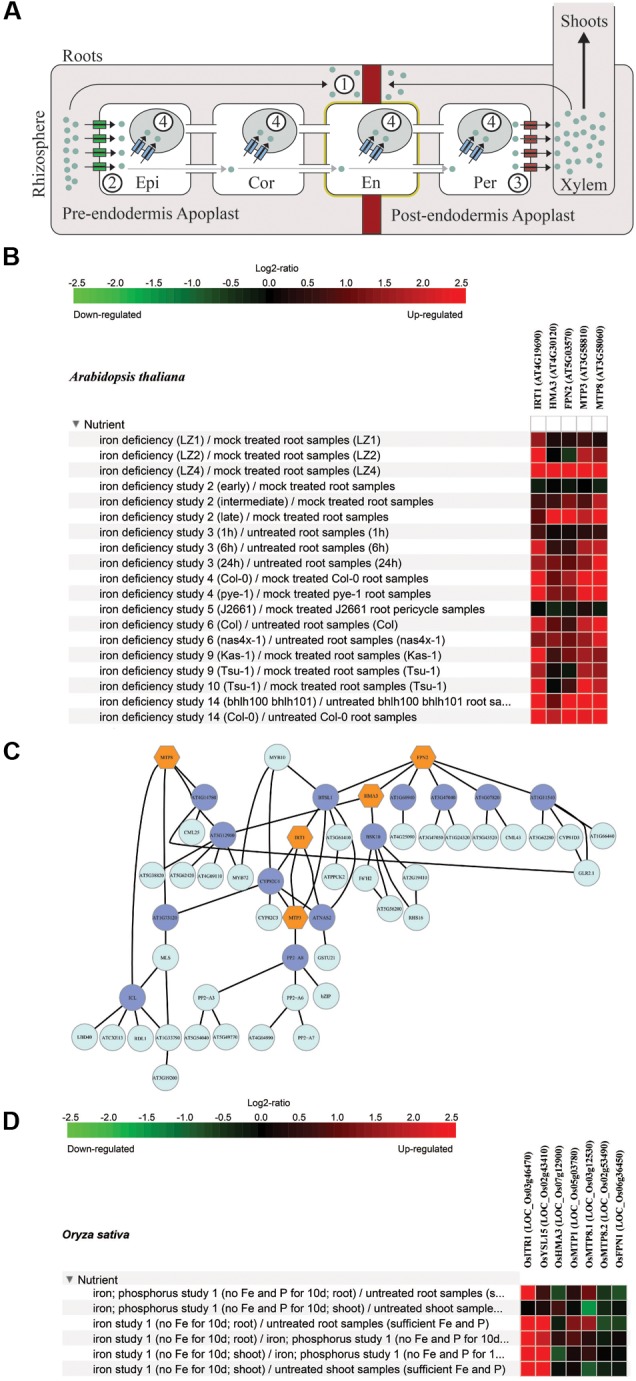
Root vacuolar compartmentalization regulating the ionome. **(A)** Checkpoints of ion radial movement within roots. (1) Endodermal diffusion barriers block ions entry in the apoplast connected to the xylem. (2) Influx and (3) efflux transporters control ion concentration in the root symplast. Influx and efflux transporters may also be present in plasma membranes of other cells, but are shown in epidermis and pericycle for clarity. (4) Root vacuoles can restrict symplastic movement of ions, and therefore decrease or increase their availability for xylem loading and shoot/seed translocation. Different cell layers may have distinct vacuolar repertoire for storage. Gray arrows indicate diffusion within the symplast through plasmodesmata. Epi, epidermis; Cor, cortex; En, endodermis; Per, pericycle. Red band – Casparian Strip; yellow = suberin deposition. **(B)** Data from Genevestigator showing regulation under Fe deficiency of Arabidopsis genes *AtIRT1* (AT4G19690), *AtHMA3* (AT4G30120), *AtFPN2* (At5G03570), *AtMTP3* (AT3G58810), and *AtMTP8* (AT3G58060). **(C)** Graphical visualization of the coexpressed gene network of the same genes as in **(B)** using ATTED-II (http://atted.jp/). Nodes (hexagons and circles) represent genes, while straight lines represent coexpression. Nodes in hexagonal shape and orange color represent genes of interest. Nodes in circle shape and purple color represent genes within the network which are directly connected to the genes of interest. Nodes in circle shape and light blue color represent genes which are connected to the purple circle genes. **(D)** Data from Genevestigator showing regulation under Fe deficiency of rice genes LOC_Os03g46470 (*OsIRT1*), LOC_Os02g43410 (*OsYSL15*), LOC_Os07g12900 (*OsHMA3*), LOC_Os05g03780 (*OsMTP1*), LOC_Os03g12530 (*OsMTP8.1*), LOC_Os02g53490 (*OsMTP8.2*), and LOC_Os06g36450 (not yet characterized, but most similar gene to *AtFPN1*/*AtFPN2*; named *OsFPN1*).

Roots are the primary sites of nutrient absorption and as such they must carefully control elemental uptake. This is accomplished via selective transporters at the plasma membrane of root cells at the epidermal and cortical cell layers. Root cells have their cytoplasm connected by plasmodesmata, membrane-lined channels that cross cell walls and allow diffusion of solutes between adjacent cells. The continuum cytoplasm-plasmodesmata of several cells make up the symplast (Rutschow et al., 2011). Once a molecule crosses an epidermal or cortical cell plasma membrane, it can move radially from the external layers into the internal stele and reach the pericycle by diffusion. The next step is xylem loading, which is also dependent on membrane selective transporters that efflux nutrients out of the symplast. Thus, the symplastic route depends on the coordination between influx transporters at the external root cell layers, efflux transporters at the internal cell layers, and diffusion between cells that are symplastically connected (Miwa and Fujiwara, 2010). Indeed, influx and efflux transport systems characterized to date were shown to be important for the control of elemental concentrations in the xylem sap and consequently in the shoots (**Figure 1A**; Miwa and Fujiwara, 2010; Sasaki et al., 2016).

Solutes from the rhizosphere can also move radially into root tissues penetrating extracellular spaces between cells and in cell walls, comprising the apoplast. However, at the endodermal cells, a lignin band deposited in anticlinal cell walls, the Casparian Strip, provides an extracellular barrier to prevent free diffusion into the stele through the apoplast (**Figure 1A**; Naseer et al., 2012). This cell wall modification interrupts the apoplastic communication from the external cell layers into the stele, thus making selective nutrient transport into the symplast necessary for ions to reach the xylem. Since endodermal cells contact both the external (connected to the rhizosphere) and internal (connected to the xylem) apoplast compartments, they are crucial to root nutrient uptake (Geldner, 2013; Barberon et al., 2016).

The composition of the Casparian Strip bands and the mechanisms and genes involved in its formation during endodermal development are being dissected in detail in the model species *Arabidopsis thaliana* (Geldner, 2013). It was shown that Casparian Strips are actually made of lignin, not suberin (Naseer et al., 2012). Recently, it was demonstrated that suberin deposition on endodermal cell surfaces in response to nutritional stress could block access of apoplast solutes to plasma membranes, therefore making absorption by the epidermis and cortical cells necessary (Barberon et al., 2016). Thus, it is expected that changes in Casparian Strip bands and suberin lamellae deposition would result in altered radial nutrient movement in roots and modified access to the xylem and shoot translocation. Indeed, changes in Casparian Strip porosity results in leakage of nutrients from one apoplastic compartment to another, which changes xylem sap concentrations and consequently perturbs the shoot ionome (Hosmani et al., 2013; Kamiya et al., 2015; Huang and Salt, 2016). Thus, maintenance of diffusional barriers in the root apoplast is important for controlling root-to-shoot translocation of nutrients.

## Root Cell Vacuoles as Checkpoints for Metal Diffusion in the Symplast and Root-To-Shoot Translocation in Arabidopsis: the Fe Deficiency Example

With proper apoplast diffusional barriers and the consequent symplastic control of absorption, the rate of uptake from the soil and xylem loading presumably determines the concentration of a given element and the amount of root-to-shoot translocation. However, root vacuoles also control nutrients and trace elements concentrations in the root symplast (**Figure 1A**). Studies in Arabidopsis have shown that specific vacuolar transporters expressed in roots perform vacuolar compartmentalization, which can impact xylem loading and root-to-shoot translocation. Loss-of-function of these transporters result in higher translocation of respective elements to shoots, presumably due to increased element availability in the root symplast for efflux into the xylem (Arrivault et al., 2006; Morrissey et al., 2009).

A striking example where vacuolar compartmentalization for multiple elements is part of a coordinated response, in which vacuoles detoxify elements that increase their concentrations due to excessive uptake, is observed during Fe deficiency response in Arabidopsis (**Figures 1B,C**). The classical Fe acquisition mechanism (reduction strategy, or strategy I) includes rhizosphere acidification by an H^+^-ATPase, Fe^3+^ reduction to Fe^2+^ by a membrane-bound, extracellular-facing reductase protein, and Fe^2+^ uptake by the high affinity transporter AtIRT1 (Brumbarova et al., 2015). AtIRT1 has broad specificity, being able to transport other divalent metals, such as Zn^2+^, Mn^2+^, Co^2+^, Cd^2+^, and Ni^2+^ (Korshunova et al., 1999; Barberon et al., 2014), which are potentially harmful. Indeed, increased concentrations of Zn, Mn, Co, and Cd in Arabidopsis shoots are part of the ionomics profile associated with physiologically Fe deficient plants, even if Fe concentration is not affected (Baxter et al., 2008). Recent work showed that non-Fe metals regulate AtIRT1 localization at the plasma membrane, which suggests that plants must balance Fe and other metal uptake through AtIRT1 under low Fe for optimal nutrition (Barberon et al., 2014).

This observation indicates that AtIRT1 is the main route of entry for these metals, which transiently accumulate in roots of Fe deficient plants. The vacuolar transporters AtMTP3, AtMTP8, AtFPN2, and AtHMA3, which are, respectively, Zn, Mn, Co/Ni, and Cd/Zn transporters (Arrivault et al., 2006; Schaaf et al., 2006; Morel et al., 2009; Morrissey et al., 2009; Eroglu et al., 2016), are coordinately up regulated upon Fe deficiency, presumably in order to decrease local high concentrations in the root symplast (**Figures 1B,C**; Buckhout et al., 2009; Thomine and Vert, 2013). Consequently, their activity can reduce metal accumulation in shoot tissues. Therefore, the action of vacuolar transporters in compartmentalization of metals into root vacuoles indirectly control the shoot ionome, indicating that root vacuoles are a checkpoint for metal movement into the xylem and can fine-tune the accumulation of essential but/or potentially toxic elements in shoots.

Interestingly, both AtFPN2 and AtHMA3 were shown to be involved in natural variation of Co and Cd shoot concentrations, respectively (Morrissey et al., 2009; Chao et al., 2012). Accessions harboring an insertion in the coding sequence of *AtFPN2*, which results in a truncated version of the protein, were hypersensitive to Co and Ni, and had increased concentrations of Co in shoots. The increased Co accumulation was more pronounced in conditions of low Fe availability, indicating that AtIRT1 uptake and AtFPN2 vacuolar compartmentalization work in concert to control Co movement in the symplastic xylem loading and root-to-shoot translocation (Morrissey et al., 2009). Regarding *AtHMA3*, a non-functional allele is present in several accessions of Arabidopsis, resulting in higher Cd concentration in shoots (Chao et al., 2012). *AtHMA3* allele variation was considered the primary determinant of Cd concentration variation in shoots of Arabidopsis multiple accessions (Chao et al., 2012). These data suggest that vacuolar sequestration in roots might be important not only to general metal detoxification, but that fine tuning of detoxification could be involved in local adaptation of distinct genotypes within a species. Therefore, allele diversity of root vacuolar transporters might help to explain natural variation in the shoot ionome.

## Root Vacuolar Compartmentalization Under Fe Deficiency in Rice

There is little evidence for a conserved mechanism during Fe deficiency response in other species than Arabidopsis, although some of the orthologous genes are also up-regulated by Fe deficiency (**Figure 1D**). In rice, the best model species for monocots, Fe deficiency induces the combined strategy (with elements from both classical strategies I and II), which up-regulates OsIRT1 (Ricachenevsky and Sperotto, 2014). However, evidence that OsIRT1 has broad specificity is still lacking, although over-expression of *OsIRT1* leads to increased Fe, Zn, and Cd concentrations in rice plants (Lee and An, 2009). In rice, the MTP group 1 clade has only one member, named *OsMTP1* (Ricachenevsky et al., 2013). OsMTP1 has been suggested to detoxify Zn into vacuoles as part of basal Zn tolerance, resembling the AtMTP1 function (Menguer et al., 2013; Ricachenevsky et al., 2015). Thus, an *AtMTP3*-like gene (i.e., with a function to detoxify high Zn under Fe deficiency) might be lacking in rice. Still, OsMTP1 may be somewhat involved in the Fe deficiency response, since expression data indicates it might be up-regulated in rice roots under low Fe conditions (**Figure 1D**).

The rice ortholog of AtHMA3, named OsHMA3, has a role in Cd vacuolar detoxification in roots. Positional cloning and natural accession screening has shown that *OsHMA3* is the causative gene of variation in Cd shoot concentrations (Miyadate et al., 2011; Yan et al., 2016). Conversely, over-expression of *OsHMA3* resulted in increased Cd tolerance, with Cd concentrations increasing in roots and decreasing in shoots (Sasaki et al., 2014). These results show that OsHMA3 performs a similar role as AtHMA3, and that both are targets for variation in Cd concentrations within each species.

There are two *AtMTP8* orthologous genes in rice: the duplicated gene pair *OsMTP8.1* and *OsMTP8.2*. Both proteins are localized at on the vacuole and are involved in Mn tolerance, similar to what is described for AtMTP8. However, decreased expression or loss-of-function of both transporters results in lower Mn concentrations in roots, but not in shoots, indicating that they may work differently than AtMTP8 regarding its role in controlling shoot translocation (Chen et al., 2013; Takemoto et al., 2017). Interestingly, OsMTP8.1 seems to be up-regulated by Fe deficiency, similar to AtMTP8 (**Figure 1D**).

## Root Vacuolar Compartmentalization Impacts the Grain Ionome

Recent work has clearly shown that root vacuolar transporters can also affect mineral accumulation in grain. In rice, *OsHMA4* is the causative gene of high grain Cu phenotype found in some accessions. Loss-of-function or natural variants with decreased OsHMA4 function result in increased Cu concentration in shoots and grains, as well as decreased concentration in roots and in root cell sap. Thus, OsHMA4 detoxifies Cu into root vacuoles, which decreases Cu translocation to shoots and grains (Huang et al., 2016). Similarly, the rice tonoplast-localized OsABCC1 transporter was shown to detoxify arsenic (As) by transporting As(III)-phytochelatin into vacuoles. The *osabcc1* mutants have increased As sensitivity and As accumulation in grains (Song et al., 2014). In Arabidopsis, the duplicated pair *AtABCC1*/*AtABCC2* also has a similar role (Song et al., 2010), again indicating that there is conservation of function in distantly related species. Moreover, *OsHMA3* natural variation was clearly linked to high/low Cd in rice grains (Yan et al., 2016).

The vacuolar iron transporter (VIT) family also deserves attention. In Arabidopsis, the *AtVIT1* gene is key for correct distribution of Fe within seeds (Kim et al., 2006). Interestingly, rice has two genes, *OsVIT1* and *OsVIT2*, which are involved in Fe storage in flag leaves. Intriguingly, high Fe (a common condition in lowland rice) up regulates *OsVIT2* in roots, indicating that rice plants might have a mechanism to avoid Fe toxicity using root vacuolar compartmentalization. Moreover, OsVIT1/OsVIT2 also seem to regulate Fe distribution in seeds (Zhang et al., 2012), and endosperm-specific over-expression of the orthologous wheat gene *TaVIT2* results in increased Fe content in wheat grains (Connorton et al., 2017).

Based on that, we expect that Arabidopsis mutants and/or natural variants with weak alleles for *AtMTP3*, *AtMTP8*, *AtFPN2*, and *AtHMA3* would have higher concentrations of their respective substrates in seeds. Indeed, AtMTP3-RNAi plants show increased Zn concentrations in whole inflorescences and siliques, and marginal increase in seeds (Arrivault et al., 2006). Under Mn sufficient conditions, loss-of-function *mtp8* mutants and WT showed similar or slightly increased Mn concentration in seeds, whereas under Mn deficient conditions the mutants had decreased concentration compared to WT (Chu et al., 2017; Eroglu et al., 2017). These results suggest that in the presence of Mn, the lack of vacuolar root transporter allow more Mn to be translocated to seeds, compensating for decreased sink strength for Mn in the *mtp8* mutant, which is apparent under Mn deficient conditions (Eroglu et al., 2017). It would be interesting to have data on Mn and Zn concentration in seeds of *mtp8* mutants and AtMTP3-RNAi plants grown under high concentration of each element. Moreover, mutants or natural variants for AtHMA3 and AtFPN2 did not have their seed metal concentration evaluated (Morel et al., 2009; Morrissey et al., 2009; Chao et al., 2012), and no data for seeds of these plants is available in the Ionomics Hub database^1^.

It is important to note that available evidence indicates that vacuolar compartmentalization is one of the checkpoints controlling the seed ionome, but by no means the only one. Depending on the element and its chemical speciation, other transporters expressed throughout the plant could contribute to the regulation of translocation to developing seeds (Sperotto et al., 2012; Punshon et al., 2018). An interesting example is highlighted by natural variation in AtHMA3, which is linked to species-wide Cd concentration variation in leaves but not to Zn and cobalt (Co) variation to the same extent, despite being able to transport both (Morel et al., 2009; Chao et al., 2012). One possibility is that elements such as Zn might be more tightly regulated, and thus variation in vacuolar transporter activity in roots might be compensated by other transporters (Chao et al., 2012). Thus, it remains to be tested the extent to which vacuolar compartmentalization is important to seed accumulation, which elements are most impacted, the transporters involved in this regulation, and how that varies in different species.

## Conclusion

Biofortification of grains has been a long sought goal on the plant nutrition field, especially for Fe and Zn, the two most commonly lacking minerals in the human diet (Sperotto et al., 2012; Ricachenevsky et al., 2015). Arsenic is also a problem in rice, since it can accumulate in grains to harmful levels for human consumption (Punshon et al., 2017). Root vacuolar compartmentalization works in concert with other checkpoints to control elemental translocation to the shoots and, consequently, to the grains. Therefore, we should expect that mutants and/or accessions with loss-of-function alleles coding root vacuolar transporters have increased concentrations of the respective elements in the xylem sap, and potentially increased concentrations in seeds.

We conclude that (1) tonoplast-localized root transporters can fine-tune symplastic concentrations of ions, together with apoplastic barriers and influx/efflux transporters; (2) plants are likely to tolerate changes in vacuolar storage capacity without strong changes in phenotype, since natural variation harbors loss-of-function alleles; and (3) orthologous genes in distantly related species might be hotspots of genetic variation (Morrissey et al., 2009; Chao et al., 2012; Huang et al., 2016; Yan et al., 2016). Thus, vacuolar transporters in roots are good candidates to search for interesting alleles and for engineering both shoot and seeds’ ionome for biofortification and nutritional quality.

## Author Contributions

FR, JF, and RS wrote the manuscript. FR and AdAJ drew figures and presentation of previously published public data. All authors approved the final manuscript.

## Conflict of Interest Statement

The authors declare that the research was conducted in the absence of any commercial or financial relationships that could be construed as a potential conflict of interest.

## Footnotes

1 www.ionomicshub.org
